# Sex-based heterogeneity in response to first-line immunotherapy plus chemotherapy in advanced esophageal squamous-cell carcinoma: a meta-analysis

**DOI:** 10.3389/fimmu.2026.1784688

**Published:** 2026-02-27

**Authors:** Kai Yang, Wei Shang, Lu Wang, Jianzhong He, Zhigang Zuo, Quankai Dai, Jialing Liu, Langhong Zeng, Yuntian Yang, Fengjun Cao, Yuandong Yu, Guoxing Wan

**Affiliations:** 1 Department of Emergency, Renmin Hospital, Hubei University of Medicine, Shiyan, China; 2 Department of Orthopedics, Renmin Hospital, Hubei University of Medicine, Shiyan, China; 3 Institute of Precision Cancer Medicine and Pathology, School of Medicine, Jinan University, Guangzhou, China; 4 Department of Pathology, The fifth Affiliated Hospital, Sun Yat-Sen University, Zhuhai, China; 5 Department of Oncology, Renmin Hospital, Hubei University of Medicine, Shiyan, China

**Keywords:** chemotherapy, difference, esophageal squamous-cell carcinoma, immune checkpoint inhibitors, sex

## Abstract

**Objective:**

Amid acknowledged sex-based disparities in immune system response, the effect of patients’ sex on the efficacy of immune checkpoint inhibitors (ICIs) treatment remains inconsistent across cancers, and even inconclusive in esophageal squamous-cell carcinoma (ESCC). We conducted a systematic review and meta-analysis to assess the sex-based heterogeneity in response to first-line immunotherapy in advanced ESCC.

**Methods:**

PubMed, Web of Science, Cochrane Library and Embase were searched from inception to December 1st, 2025 to retrieve randomized controlled trials (RCTs) investigating the efficacy of first-line immunotherapy plus chemotherapy versus chemotherapy alone for advanced ESCC. The studies reporting sex-stratified outcomes for overall survival (OS) with or without progression-free survival (PFS), were eligible for inclusion. Pooled hazard ratios (HRs) and 95%CI were calculated separately in men and women using a random-effects model, and the heterogeneity between the two estimates was assessed using an interaction test.

**Results:**

Nine phase 3 RCTs, reporting on 4591 men (85.6%) and 773 women (14.4%), were included. An OS benefit of immunotherapy was found for both men (HR, 0.70; 95%CI, 0.65-0.75) and women (HR, 0.71; 95%CI, 0.58-0.87) in the overall population and in the PD-L1-positive subgroup (HR for men: 0.66, 95%CI, 0.55-0.80; HR for women: 0.48, 95%CI, 0.30-0.78). Similarly, the PFS benefit was found for both men (HR, 0.59; 95%CI, 0.54-0.63) and women (HR, 0.58; 95%CI, 0.46-0.74) in the overall population. Random-effects meta-analysis demonstrated no statistically significant study-level differences in response to immunotherapy between the sexes in the overall population (OS, I^2^ = 14%; P = 0.94; PFS, I^2^ = 18%; P = 0.95) as well as in the PD-L1-positive subgroup (PFS, I^2^ = 0%; P = 0.24).

**Conclusion:**

First-line immunotherapy plus chemotherapy can improve OS and PFS in advanced ESCC for both men and women. No evidence was found to support an association of sex with the efficacy of immunotherapy plus chemotherapy.

## Introduction

Immune checkpoint inhibitors (ICIs) in combination with chemotherapy have demonstrated higher efficacy than chemotherapy alone against various solid cancers (1). Given the biological basis of ICIs to enhance antitumor immunity, the benefit from ICIs treatment differs in patients with different immunologic background (2). Substantial sex differences in the immunologic response to both foreign and self-antigens were described (3), with women typically mounting stronger innate and adaptive immune responses than men (4). These differences may account for higher prevalence of systemic autoimmune diseases, greater vaccine efficacy, and lower severity and prevalence of many infections in women (5). In oncology, variations in immune response are believed to contribute to the observed differences in prevalence and mortality across different cancers (6–9). Moreover, the hormonal effects on the activity of programmed cell death 1 (PD-1)/programmed cell death 1 ligand 1 (PD-L1) pathway have been evidenced in animal models (10–12). Thus, it is plausible to hypothesize that patient sex may influence responses to immunotherapy.

Recently, increasing literature points to sex differences in survival benefits related to immunotherapy. The meta-analysis by Conforti et al. evolving 20 randomized controlled trials (RCTs) reported that men derived greater benefit from ICIs compared with women in advanced cancers (5). Similar finding with regard to non-small cell lung cancer immunotherapy was presented in the meta-analysis by Liang et al (13). However, conflicting results from other meta-analyses were reported by Wallis et al. and Yang et al. who demonstrated no statistically significant association between patient sex and the magnitude of benefit from immunotherapy in advanced cancers (2, 6). To our knowledge, the specific effect of sex on the efficacy of immunotherapy in patients with esophageal squamous-cell carcinoma (ESCC) remains inconclusive.

Although combination of platin/fluoropyrimidine-based systemic chemotherapy with ICI is the novel standard of care for advanced ESCC in the first-line setting (14), optimal care requires research to select patients who will benefit from immunotherapy. Now that the results of several RCTs with immunotherapy for advanced ESCC in the first-line setting have become available, we performed a systematic review and meta-analysis that examines the association of patient sex with survival benefit of immunotherapy.

## Methods

This study was performed in adherence with the Preferred Reporting Items for Systematic Reviews and Meta-analyses (PRISMA) guidelines (15). The study protocol was registered with PROSPERO (CRD42024531327).

### Data Sources and Searches

We searched PubMed, Web of Science, Cochrane Library and Embase from inception to December 1st, 2025, to identify phase 2 and 3 RCTs that examined immunotherapy-chemotherapy combinations compared with chemotherapy alone for advanced ESCC in the first-line setting. Two investigators (GW and YY) independently performed the database search. The search terms included “PD-1”, “programmed death receptor 1”, “PD-L1”, “programmed death ligand 1”, “nivolumab”,”pembrolizumab”, “avelumab”, “durvalumab”, “atezolizumab”. The search strategy is detailed in **Supplementary Table 1**. References from review articles, editorials, and included studies were reviewed and cross-referenced to ensure completeness. The language of publication was restricted to English with no limitation on publication year. Single-arm phase 1 and 2 trials without RCT design (ie, nonrandomized trials) were also excluded. To be eligible, studies had to meet all of the following criteria: 1) RCT examining the combination of immunotherapy with chemotherapy against chemotherapy alone, and 2) studies including previously untreated locally advanced, metastatic or recurrent ESCC, 3) data available on hazard ratio (HR) and confidence interval (CI) for overall survival (OS) with or without progression-free survival (PFS), according to patients’ sex subgroup. Studies that compared immunotherapy plus chemotherapy with chemotherapy alone in the second-line or post-line setting were excluded. Analyses that examined a single ICI or ICI combinations compared with chemotherapy alone were also included.

### Study selection and data extraction

Two investigators independently reviewed the list of retrieved articles to choose potentially relevant articles, and disagreements about particular studies were discussed and resolved with the consensus of all investigators. We included only the most recent and complete report of the RCT when duplicate publications were identified. Study characteristics, including first author, name of study, number of patients, age distribution, sex distribution, type of ICI and chemotherapy used and follow-up distribution were extracted. Additionally, outcome information, including HRs and 95%CIs for PFS and/or OS in the overall population, and HRs with CIs according to patients’ sex and PD-L1 expression, were abstracted. A risk-of-bias assessment was conducted using the Cochrane Collaboration tool for assessing risk of bias.

### Data synthesis and statistical analysis

The primary endpoint was the difference in efficacy of immunotherapy, which was measured in terms of the ratio of the HR for progression or death in the intervention arm (immunotherapy plus chemotherapy) compared with those in the control arm (chemotherapy alone) reported in men, to the same HR reported in women.

Considering the clinical heterogeneity inherent in the data, random-effects model weighted by the inverse of variance was used in all meta-analyses to calculate the pooled HRs. The between-study heterogeneity was assessed by Q test with the DerSimonian-Laird method, and the heterogeneity was also quantified using I^2^ statistics. To assess the differences between the sexes in each study, logHR was calculated, which was further assessed by the null hypothesis as previously described. Briefly, the null hypothesis was set as that the difference of the survival benefit of immunotherapy between women and men is zero. The null hypothesis was then tested using the following approach: first, we calculated a study-specific interaction HR (95%CI) in each study from the ratio of the reported HRs (95%CIs) in men and in women; second, we pooled these study-specific interaction HRs (95%CIs) across trials using a random-effects model. Given the predictive value of PD-L1 expression in efficacy of immunotherapy, the sex-based variations were also examined in PD-L1-positive study participants. The definition for PD-L1-positive was that immunohistochemistry-based tumor cell proportion score (TPS) or combined positive score (CPS) is greater than 1%. However, we solely assessed the sex-based variations in terms of OS benefit in PD-L1-positive subpopulations because no available data concerning PFS were provided. A pooled HR ratio estimate <1 indicates a greater survival benefit in men, and >1 a greater survival benefit in women. All reported P values were 2-sided, and a P value less that 0.05 was considered to indicate statistical significance. All meta-analyses were performed using Review Manager Software (version 5.2).

## Results

### Literature search results

**Figure 1:**
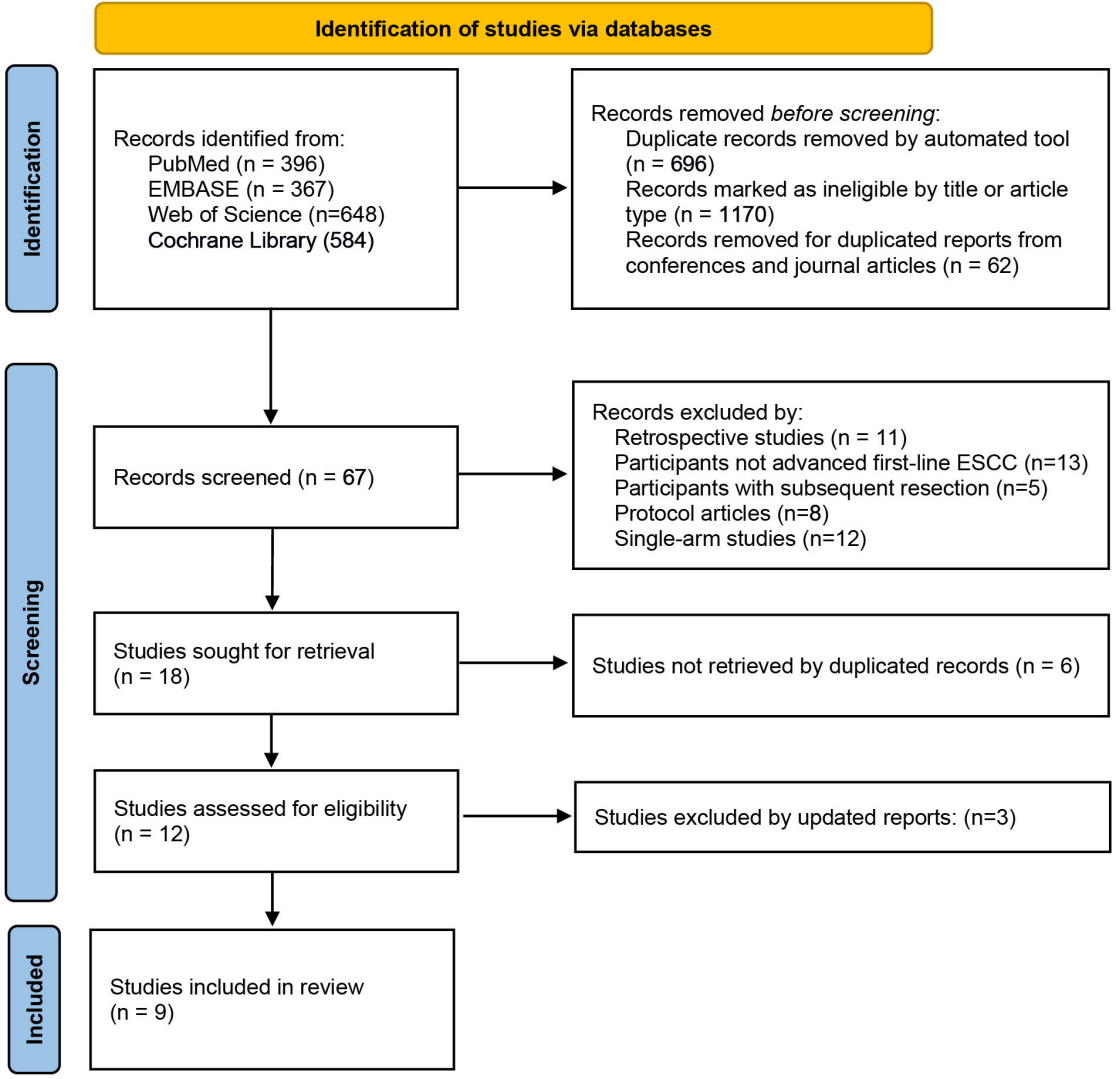
PRISMA diagram.

Online database searching retrieved 1411 publications, of which 62 were reviewed in full. Totally, nine eligible RCTs were included in the present meta-analysis (**Figure 1**). All nine trials had available data on the evaluation of survival benefit of immunotherapy, stratified by sex, that compared immunotherapy plus chemotherapy with chemotherapy alone, with an HR for OS, and seven trials with an HR for PFS. Additionally, two trials ASTRUM-007 and CheckMate-648 reported the OS results stratified by sex in the PD-L1-positive subgroup.

### Characteristics of identified trials

The main characteristics of the 9 trials are listed in **Table 1**. In total, 5364 patients were included, of which 4591 (85.6%) were men and 773 (14.4%) were women. All studies enrolled patients within the past decade, and most trials were published in the past 3 years. All trials were phase 3, and the study participants in all trials were ESCC except for one trial KEYNOTE-590 including 73% squamous cell carcinoma and 27% adenocarcinoma. Eight trials (ASTRUM-007, CheckMate-648, GEMSTONE-304, JUPITER-06, KEYNOTE-590, ORIENT-15, RATIONALE-306, SKYSCRAPER-08) were conducted in locally advanced or metastatic settings (16–23), while one trial (ESCORT-1^st^) was conducted in the metastatic setting only (24). The immunotherapy was anti-PD-1 ICIs in eight trials and anti-TIGIT plus anti-PD-L1 ICI in one trial, and the chemotherapy regimen was cisplatin plus fluorouracil (PF) in four trials (ASTRUM-007, CheckMate-648, GEMSTONE-304, KEYNOTE-590), paclitaxel plus cisplatin (TP) in three trials (JUPITER-06, ESCORT-1^st^, SKYSCRAPER-08), and PF/TP in two trials (ORIENT-15, RATIONALE-306). The PD-L1 expression was not restricted for patient enrollment in eight trials, whereas only patients with PD-L1-positive expression were eligible in ASTRUM-007 study. The median age of patients ranged from 62 to 65 years across all studies, and median follow-up ranged from 10.8 months to 58.8 months while three studies did not report median follow-up in their publications. Overall, all nine studies included evaluated OS and PFS as dual primary endpoints, and demonstrated clinically meaningful survival benefit for patients who received immunotherapy plus chemotherapy compared with chemotherapy alone. However, only one study RATIONALE-306 showed a significant OS advantage for female patients in the subgroup analysis, whereas this advantage was revealed by all studies for male patients. Meanwhile, all seven studies demonstrated a significant PFS advantage from immunotherapy among men, while only three of seven studies showed this advantage among women.

**Table 1:** Characteristics and outcomes of the 9 trials included in the meta-analysis.

Trial name	Intervention (No.)	Control (No.)	Age, median for arms (Range or IQR),y	Follow-up for arms, Median(Range or IQR), month	Sex, no. (%)		Overall survival HR (95% CI)		Progression-free survival HR (95% CI)	
			Int./Con.	Int./Con.	Men	Women	Men	Women	Men	Women
ASTRUM-007*	Serplulimab+PF (368)	Placebo+PF (183)	64(57-68)/64(57-68)	14.9(8.8-19.7)/15.0(9.4-19.9)	470 (85.3)	81 (14.7)	0.67 (0.51-0.88)	0.47 (0.21-1.03)	0.59 (0.46-0.75)	0.41 (0.19-0.85)
CheckMate 648*	Nivolumab+PF (321)	PF (324)	64(40-90)/64(26-81)	39.4(28.8-56.6)/39.6(29.0-55.9)	528 (81.9)	117 (18.1)	0.79 (0.66-0.96)	0.93 (0.61-1.43)	NR	
ESCORT-1st	Camrelizumab+TP (298)	Placebo+TP (298)	62(56-66)/62(56-67)	NR(24.0-NR)	523 (87.8)	73 (12.2)	0.71 (0.59-0.87)	0.59 (0.33-1.05)	0.54 (0.45-0.66)	0.47 (0.26-0.86)
JUPITER-06	Toripalimab+TP (257)	Placebo+TP (257)	63(25-75)/62(40-74)	NR	437 (85.0)	77 (15.0)	0.50 (0.36-0.70)	1.40 (0.60-3.28)	0.51 (0.40-0.66)	0.96 (0.53-1.75)
KEYNOTE-590	Pembrolizumab+PF (373)	Placebo+PF (376)	64(28-94)/62(27-89)	58.8(49.2-70.6)	625 (83.4)	124 (16.6)	0.70 (0.59-0.82)	0.80 (0.55-1.16)	0.62 (0.52-0.74)	0.69 (0.46-1.03)
ORIENT-15	Sintilimab+TP/PF (327)	Placebo+ TP/PF (332)	63(57-67)/63(56-67)	16.0(12.3-19.4)/16.9(11.8-20.2)	567 (86.0)	92 (14.0)	0.64 (0.51-0.81)	0.57 (0.29-1.12)	0.56 (0.46-0.69)	0.60 (0.34-1.07)
RATIONALE-306	Tislelizumab+TP/PF (326)	Placebo+TP/PF (323)	64(26-84)/65(40-84)	NR	563 (86.7)	86 (13.3)	0.72 (0.59-0.88)	0.46 (0.24-0.85)	NR	
GEMSTONE-304	Sugemalimab+PF (358)	Placebo+PF (182)	62.5(40–75)/63(43–75)	15.7(0.1-29.8)/13.8(0.2-25.9)	472 (87.4)	68 (12.6)	0.70 (0.54-0.91)	0.83 (0.39-1.74)	0.66 (0.53-0.82)	0.63 (0.34-1.16)
SKYSCRAPER-08	Tiragolumab plus atezolizumab+TP (229)	Placebo+TP (232)	63 (57–68)/63 (57–68)	14.6(8.3-18.4)/10.5(5.9-17.2)	406 (88.1)	51 (11.9)	0.72 (0.56-0.92)	0.56 (0.28-1.11)	0.60 (0.48-0.76)	0.36 (0.20-0.67)

Con, control group; HR, hazard ratio; Int, intervention group; IQR, interquartile range; NR, not reported; PF, cisplatin plus fluorouracil; TP, taxanes plus cisplatin.

### Risk of bias

Risk-of-bias assessment with the Cochrane Collaboration tool for each trial is reported in **Supplementary Figure 1**. All studies included random-sequence generation and were at low risk reporting bias. One trial was an open-label study and thus at risk for performance bias; however, the lack of blinding is likely inconsequential as blinding or not is unlikely to affect the assessment of the primary outcome (OS). Overall, the risk of bias in the included studies was deemed to be low.

### Primary analysis for overall and progression-free survival in the total population

**Figure 2:**
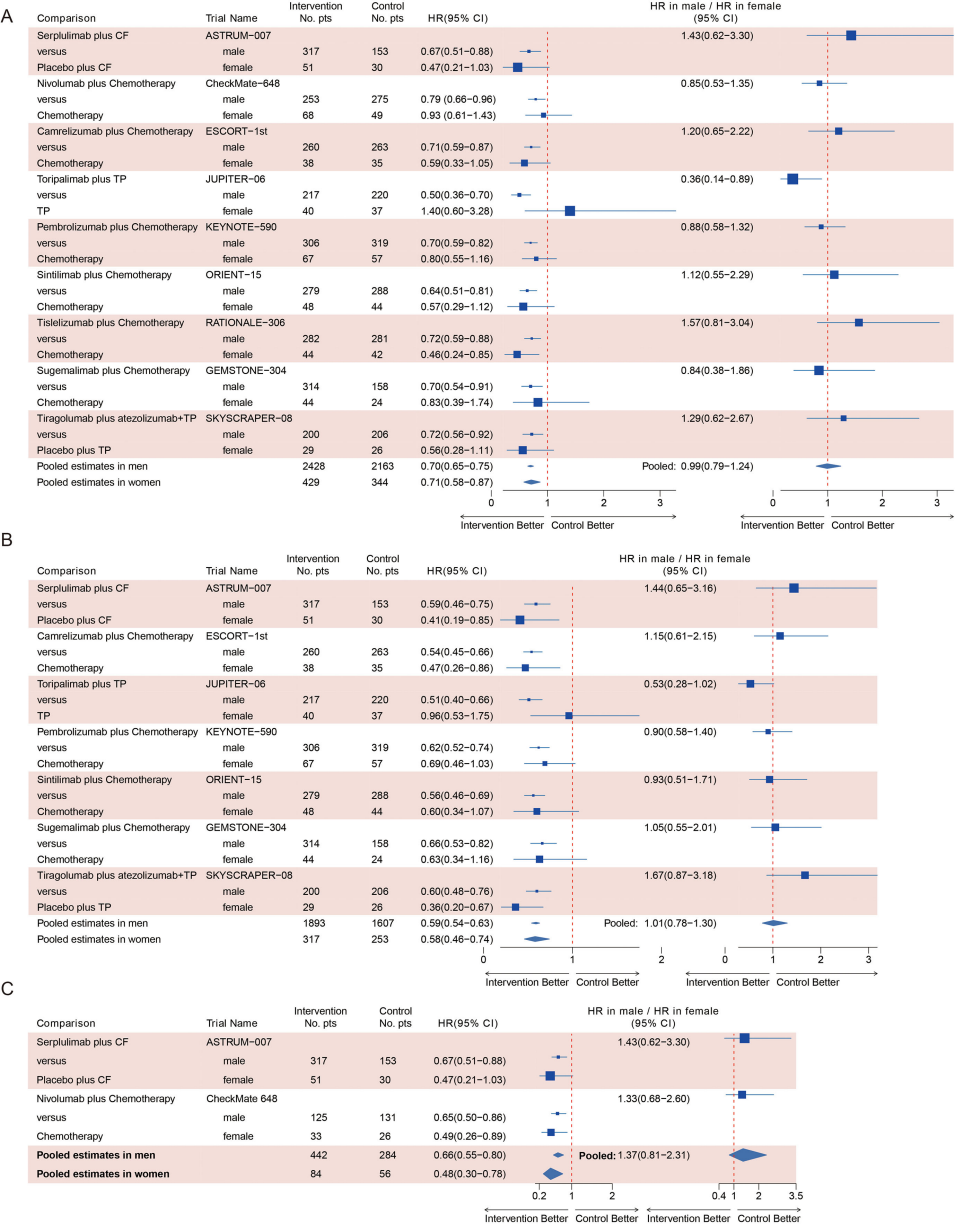
Overall survival and progression-free survival benefit of immunotherapy plus chemotherapy in advanced ESCC, by sex and PD-L1 status. **(A)** Overall survival in the total population. **(B)** Progression-free survival in the total population. **(C)** Overall survival in the PD-L1-positive subgroup.

Compared with chemotherapy alone, immunotherapy plus chemotherapy was associated with a statistically significant improvement in OS for both male (pooled HR 0.70, 95% CI = 0.65–0.75) and female patients (pooled HR 0.71, 95% CI = 0.58–0.87) (**Figure 2A**). No substantial between-study heterogeneity was observed in either male (Q = 6.39, I^2^ = 0%) or female patients (Q = 8.81, I^2^ = 9%). Similarly, such PFS advantage was also observed both in male (pooled HR 0.59, 95%CI=0.54 to 0.63) and female (pooled HR 0.58, 95%CI=0.46 to 0.74) patients treated with immunotherapy (**Figure 2B**). Also, no between-study heterogeneity was observed in either male (Q = 3.44, I^2^ = 0%) or female patients (Q = 7.08, I^2^ = 15%) when pooling PFS data. Formal interaction analysis revealed no statistically significant difference between sexes in the magnitude of OS benefit (ratio of HRs 0.99, 95% CI = 0.79–1.24; P = 0.94, I^2^ = 14%) or PFS benefit (ratio of HRs 1.01, 95% CI = 0.78–1.30; P = 0.95, I^2^ = 18%) (**Figures 2A, B**).

### Analysis for overall survival in the PD-L1-positive subgroup

As for PD-L1-positive subpopulation, two studies (ASTRUM−007 and CheckMate-648) enrolling 866 patients reported data on HR for death according to patients’ sex. Among the patients, 726 were male (83.8%) and 140 were female (16.2%). The statistically significant OS advantage of immunotherapy plus chemotherapy over chemotherapy alone was found both in male (HR 0.66, 95%CI=0.55 to 0.80; **Figure 2C**) and female (HR 0.48, 95%CI=0.30 to 0.78; **Figure 2C**) patients. There was no statistically significant difference in OS from immunotherapy between men and women (HR 1.37, 95%CI=0.81 to 2.31; P = 0.24, I^2^ = 0%, **Figure 2C**). No between-study heterogeneity was observed in all meta-analyses in the PD-L1-positive sub-population.

### Sensitivity analysis

We also conducted the sensitivity analyses to assess sex-based heterogeneity of efficacy of immunotherapy plus chemotherapy over chemotherapy alone by excluding the KEYNOTE-590 trial because 27% adenocarcinoma patients were enrolled. The results of interaction analyses showed that the pooled ratio of HRs for OS and PFS were 1.03 (95%CI=0.78 to 1.35, P = 0.84, I^2^ = 21%) and 1.05 (95%CI=0.76 to 1.44, P = 0.78, I^2^ = 29%), confirming the similar survival benefit among male and female patients receiving immunotherapy.

## Discussion

To the best of our knowledge, we conducted the first meta-analysis on sex-based heterogeneity of the magnitude of benefit in advanced ESCC patients receiving first-line immunotherapy plus chemotherapy. A substantial number of studies from various regions were included, with follow-up times of up to 58.8 months for reliable cancer recurrence detection. Our results suggest no evidence of association between sex and the level of survival benefit from immunotherapy. Furthermore, when assessing the association in PD-L1-positive subpopulation, we could not demonstrate any significant gender-specific differences in efficacy.

In terms of the association between sex and the survival benefit in advanced ESCC patients receiving first-line immunotherapy plus chemotherapy, our results were similar to those of the meta-analysis concentrating on pan-cancer by Wallis et al. (6), Lai et al. (25), Yang et al. (2), and Lai et al. (26), but different from the meta-analyses performed by Conforti et al (5), Wu et al. (27) and Trinkner et al. (28), which reported that men benefited more from immunotherapy. These conflicting results may be explained by that the sex-based heterogeneity for immunotherapy was likely distinct across cancers. Specifically, the sex-dependent magnitude of benefit from immunotherapy was found particularly obvious in melanoma and non-small-cell lung cancer but modest even absent in other cancers in the pan-cancer meta-analysis by Conforti et al (5). However, a meta−analysis concentering gastric or gastro−oesophageal junction cancer demonstrated significant OS benefit from immunotherapy in male patients but not in female patients (29), while Yanagisawa et al. demonstrated that OS benefit of first-line ICI-based combination therapy in metastatic renal cell carcinoma (mRCC) and urothelial carcinoma (mUC) was independent of gender (30). Collectively, these seemingly contradictory findings underscore a critical, unifying theme that the association between sex and immunotherapy efficacy is not a fixed biological rule, but a variable that is profoundly modulated by context. The inconsistence is not merely statistical noise but a reflection of the diverse biological and clinical landscapes across different cancers and treatment regimens, such as different tumor histotypes, lines of therapy, agents of immunotherapy, and intervention therapies.

In general, esophageal cancer occurs far more frequently in men than in women (31). However, there were no consistent results reporting a more inferior outcomes of male patients with esophageal cancer. For instance, in a population-based study to evaluate the sex differences in cancer-specific survival (CSS) for locally advanced esophageal cancer after neoadjuvant chemoradiotherapy (nCRT), Wang et al. found that male gender was independently associated with a shorter CSS (HR: 1.29, 95% CI, 1.04 -1.58) compared to female (32). In a retrospective study assessing the sex difference in survival of patients treated by surgical resection for esophageal cancer in Japan, Hidaka et al. demonstrated that long-term survival after surgical resection of the esophagus appeared to be significantly better for women than for men (31). Similarly, Noh et al. found that female patients had a more favorable survival outcomes of esophageal cancer compared to male patients among the Korean population (33). Whereas, this sex difference in survival was not observed in either esophageal adenocarcinoma (EAC) or ESCC in another population-based study by Stabellini et al (34). Despite of the discrepancy which may be explained by limited sample size and the retrospective nature of the individual study, the superiority of survival outcome in female was demonstrated in most of the studies. Of note, the survival advantage of female gender established by previous studies was mainly at the era without immunotherapy. Recently, in a retrospective study to identify biomarkers and characteristics of patients who benefit from ICI monotherapy, male gender was independently associated with better OS and PFS in ESCC compared to female (35). By contrast, women seem to respond as well as men when receiving first-line immunotherapy plus chemotherapy in our analysis, which allows us to speculate that the chemotherapy may abrogate the survival benefit of immunotherapy for female patients in the advanced setting of ESCC. Furthermore, emerging evidence suggests that tumor mutational burden (TMB) may be a key biological modulator of sex-differential responses to ICIs, with high TMB potentially equalizing efficacy across sexes (36). This mechanism could also contribute to the comparable outcomes observed in our study, assuming a relevant proportion of patients had high TMB. Given these discrepancies, the considerable effect of gender on tailoring immunotherapy in ESCC should be prospectively evaluated in future.

Another important aspect that has to be considered when evaluating different treatment outcomes between the genders may be age. After curative resection for carcinoma of the esophagus, a retrospective study involving 469 patients reported a significantly better 5-year survival for women compared to men (35% vs. 16%, p= 0.008), but this seems to be limited to patients with the age <49 years at the diagnosis (37). In a population-based study evaluating the influence of sex on the survival of patients with esophageal cancer, women were found to have longer esophageal cancer-specific survival (ECSS) than men in both metastatic esophageal cancer (MEC) and locoregional esophageal cancer (LEC) cohorts. However, when accounting for age in the squamous cell MEC cohort the ECSS advantage for women was limited to patients younger than 55 years (38). Conversely in the era of immunotherapy, a previous study found that patients with the age >60 years responded more efficiently to ICI therapy compared to those with the age <60 years (39). Similarly, a meta-analysis by Wu et al. reported an apparently larger relative benefit from ICI vs control therapy for patients aged 65 years or older than for those younger than 65 years (40). Indeed, immunosenescence-induced difference on overall immune function and immune cell subsets between younger and older populations has been well characterized (40). A previous study found that the level of FOXP3+ regulatory T cells (Tregs) in the melanomas of young mice receiving ICIs treatments was significantly higher compared to the aged mice while the CD8þ effector T-cell numbers was lower, which could result in a significant decrease in anti-tumor immune response of young mice (39). Moreover, both innate and adaptive immune responses differ between males and females at young and advanced ages, which was closely related to alterations in endocrine sex hormone system (41–43). Available data indicate that young adult females demonstrate a more reactive, inflammatory profile when compared with young adult males (44), and the immune systems of aged women appear to remain skewed toward an inflammatory phenotype, while it appears to be more moderate in aged men (45, 46). Moreover, the previous study found that estradiol, via estrogen receptorα, induces the polarization of tumor associated macrophages (TAMs) toward the immune-suppressive M2 phenotype at the expense of the anti-tumor M1 phenotype, leading to a dysfunctional cytotoxic T cell antitumor response. Male and female upon reaching a specific age experience contrasting rise and dip of estradiol, which may have a different impact on the response to immunotherapy. Additionally, sex-dependent differences in CD8^+^ T-cell phenotypes, such as activation, exhaustion, or memory status within the tumor microenvironment or peripheral blood, could further underlie disparities in immunotherapy outcomes (47). Accordingly, it is biologically plausible to speculate that the difference of age-dependent hormonal effects in female and male may have an impact on the efficacy of immunotherapy. However, the age-dependent sex difference in response to immunotherapy plus chemotherapy in advanced ESCC remains undetermined due to the lack of available data.

Gender-specific effects on survival benefit from immunotherapy might also be associated with body mass index (BMI). Sufficient data have shown that higher BMI is associated with risk of numerous malignancies including esophageal cancer, and the main mechanisms underlying this relationship include the insulin/IGF1 system, the effect of sex hormones, and adipocytokines (48). In a population-based study involving 451,500 UK Biobank participants to examine the relation of BMI to cancer incidence, BMI showed a stronger association with a higher risk of esophageal cancer in males than in females (49). Considering the role of BMI for ICI treatment outcomes, conflicting findings were revealed by previous studies. The systematic review by Indini et al. concluded that the current evidence with inconsistent findings from individual studies could not support definitely an association of BMI with survival outcomes in patients receiving immunotherapy (50). By contrast, three other meta-analyses demonstrated a positive association of high BMI with improved OS and PFS (28, 51, 52). This apparent discrepancy likely results from the methodological limitation of a qualitative systematic review, which lacks the statistical precision of quantitative meta-analyses to yield robust effect estimates. In support of the findings from the previous meta-analyses, the stratification analysis of ESCORT-1^st^ trial included in the present study showed a trend toward more survival benefit from immunotherapy plus chemotherapy in advanced ESCC patients with baseline BMI ≥ 20 kg/m² than those with baseline BMI < 20 kg/m² (HR for OS: 0.65 vs. 0.82; HR for PFS: 0.52 vs. 0.59) (24). Similarly, patients with baseline weight ≥ 60 kg appeared to benefit more than those with baseline weight < 60 kg in the ORIENT-15 trail (HR for OS: 0.59 vs. 0.67; HR for PFS: 0.51 vs. 0.59) (21). However, a consensus that whether BMI-related benefit from immunotherapy was associated with gender has not been reached with the current evidence. A multicenter retrospective study showed that overweight (BMI>25 kg/m²) was predictive of favorable survival outcome in cancer patients treated with ICIs therapy in both sex, while the predictive significance for PFS was limited to male patients (53). Similarly, a recent meta-analysis by Trinkner et al. involving 19,767 patients from 48 studies showed that overweight/obesity was significantly associated with better survival outcome in male patients but not in female patients, suggesting that body composition is associated with survival in a sex-specific manner in cancer patients undergoing ICI treatment (28). Conversely, the meta-analysis by Xu et al. involving 4090 patients from 16 studies demonstrated that obesity-related clinical benefit is independent of sex in cancer patients treated with ICIs therapy (54). The reasons accounting for the discrepancy may include small-sample effect, heterogeneous cancer populations, and different cut-off values for BMI categorization across different studies. Regretfully, the sex-dependent difference in body composition-related survival benefit from ICI treatment in ESCC patients remains undetermined due to the lack of sufficient data.

While this meta-analysis focused on sex-based heterogeneity in the context of first-line chemoimmunotherapy for advanced metastatic ESCC, the therapeutic landscape for ESCC is rapidly evolving to include multimodal, stage-adapted strategies. Notably, for patients with locally advanced ESCC, induction chemoimmunotherapy followed by definitive radiotherapy is being actively investigated and has emerged as a promising research avenue (55). A recent editorial by Ma and Baran comprehensively discussed the potential of this approach, highlighting that induction chemoimmunotherapy followed by definite radiotherapy or concurrent chemoradiotherapy can yield superior survival outcomes compared to conventional chemoradiotherapy in the locally advanced setting, with a manageable safety profile. The rationale hinges on exploiting the synergistic effects of immunotherapy and radiotherapy to remodel the tumor immune microenvironment and potentially enhance treatment responses (56). Although the present study did not include trials investigating such combined-modality regimens for locally advanced disease, this evolving paradigm underscores an important direction for future research. It will be crucial to investigate whether the absence of a significant sex-based differential benefit from immunotherapy, as observed here in the metastatic first-line setting, holds true across different disease stages (e.g., locally advanced, induction, or maintenance settings) and treatment combinations (e.g., immunotherapy concurrently or sequentially with radiotherapy). As treatment moves earlier in the disease course, patient immune status, tumor burden, and microenvironmental characteristics may differ, potentially influencing the interplay between sex and treatment efficacy. Prospective studies integrating these novel multimodal approaches should therefore consider stratified analyses by sex to confirm the generalizability of our findings and to ensure equitable and optimized treatment strategies across all patient subgroups. Nevertheless, our study demonstrated the absence of a significant sex-based differential benefit from first-line immunotherapy plus chemotherapy, providing a clear, actionable insight: patient sex should not be used as a deciding factor when considering the addition of an ICI to first-line chemotherapy in advanced ESCC. This simplifies the initial therapeutic decision, allowing clinicians to focus on other established factors such as PD-L1 expression, performance status, patient immune status, CD8 T cell phenotype, tumor burden and patient comorbidities without the need for sex-based stratification.

Several limitations should be acknowledged in the present study. First, the meta-analysis was conducted on aggregate study data of published RCTs subgroup HRs rather than individual participant data. The direct comparison would provide a more accurate result. Second, women were comprised almost less than quarter of the study population in each trial. Although the meta-analysis of such small subgroup analyses would enhance the statistical power, it may also increase the possibility of false discovery rates or even false-positive results. Third, residual confounding other than sex is predictive of survival benefit from immunotherapy plus chemotherapy in ESCC. Fourth, the OS and PFS data from some included RCTs were not the final reports, and the median follow-up varied from 10.8 months to 58.8 months, which may increase the heterogeneity between individual trials. Fourth, our search was restricted to English-language databases (PubMed, Web of Science, EMBASE, Cochrane Library) and did not include Chinese databases such as CNKI or Wanfang. Given China’s high incidence of ESCC and its active research landscape, this may introduce geographic and language bias, potentially omitting relevant investigator-initiated trials or regional studies published in Chinese. However, we included Chinese RCTs published in English in international journals, more than 80% of the enrolled patients across our analyzed studies were Chinese. Future systematic reviews in this field may benefit from multilingual search strategies and collaboration with Chinese research teams to ensure comprehensiveness. Fifth, while certain immune-related adverse events are known prognostic biomarkers for ICI therapy (57), it remains unclear whether the occurrence of specific adverse events interacts with patient sex to predict survival outcomes in the setting of advanced ESCC due to a lack of primary, patient-level data. This represents an important avenue for future research. Finally, sex-based responses to ICIs and chemotherapy are driven by complex interactions between sex hormones, TMB, genetics (such as NOTCH1 mutation), gut microbiome (such as *Firmicutes and Bacteroidetes*), and body composition, leading to distinct immunological and pharmacokinetic profiles (58, 59). The robustness of our results may be affected by the interaction effects between sex and these confounding factors in the absence of stratification analyses.

## Conclusion

In this contemporary meta-analysis of all available RCTs, our finding suggested that patients with different sex could derive a similar magnitude of survival benefit from the first-line immunotherapy plus chemotherapy in advanced ESCC. We found no evidence to support the consideration of sex-dependent difference when deciding whether to offer first-line immunotherapy plus chemotherapy in advanced ESCC.

## Data Availability

The original contributions presented in the study are included in the article/**Supplementary Material**. Further inquiries can be directed to the corresponding author.

## Supplementary material

Supplementary Figure 1 Risk of bias assessment for included studies.
