# Supplementary figures and images for: Sex-based heterogeneity in response to first-line immunotherapy plus chemotherapy in advanced esophageal squamous-cell carcinoma: a meta-analysis
Source: Front Immunol. 2026 Feb 27;17:1784688. doi: 10.3389/fimmu.2026.1784688 (PMC12982060; doi:10.3389/fimmu.2026.1784688)

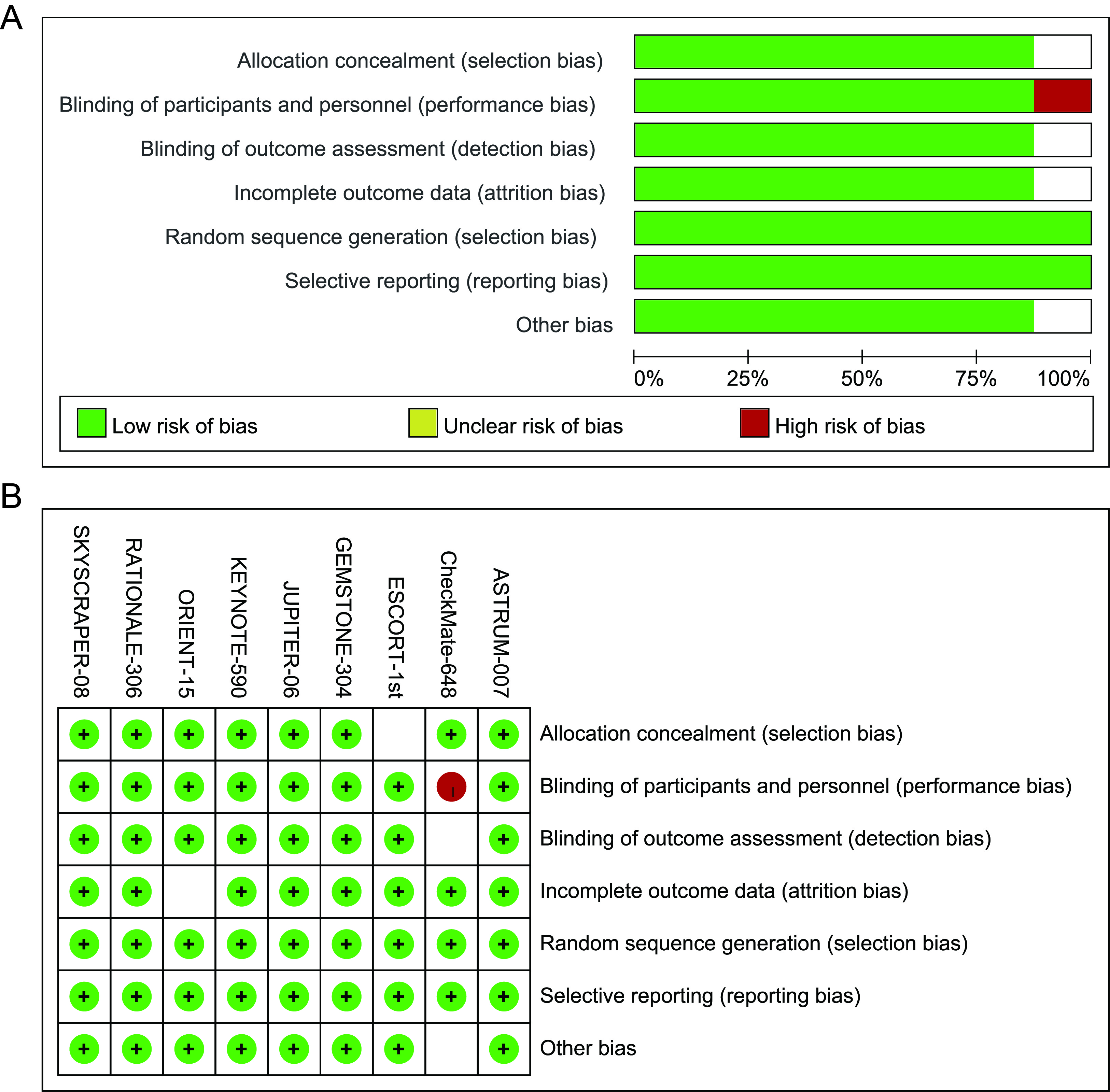

Supplement: Supplementary Figure 1 — Risk of bias assessment for included studies. [file Image1.jpeg]
