# Supplementary material for: Sex-based heterogeneity in response to first-line immunotherapy plus chemotherapy in advanced esophageal squamous-cell carcinoma: a meta-analysis
Source: Front Immunol. 2026 Feb 27;17:1784688. doi: 10.3389/fimmu.2026.1784688 (PMC12982060; doi:10.3389/fimmu.2026.1784688)
Supplement: Supplementary file 2 [file Table1.docx]

**Table S1. Search strategy for potential studies.**

**PubMed**

#1 "Esophageal Neoplasms"[MeSH Terms]

#2 "esophageal neoplasm"[Title/Abstract] OR "cancer of esophagus"[Title/Abstract] OR "esophageal cancer"[Title/Abstract] OR "esophageal cancers"[Title/Abstract] OR "cancer of the esophagus"[Title/Abstract] OR "esophagus cancer"[Title/Abstract] OR "esophagus cancers"[Title/Abstract] OR "ESCC"[Title/Abstract] OR "esophageal squamous cell carcinoma"[Title/Abstract]

#3 #1 OR #2

#4 (((advanced[Title/Abstract]) OR (metastatic[Title/Abstract])) OR (unresectable[Title/Abstract])) OR (inoperable[Title/Abstract])

#5 #3 AND #4

#6 "Immune Checkpoint Inhibitors"[Mesh]

#7 (((((((((((((((((((((((((((((((immune checkpoint[Title/Abstract]) OR (immune checkpoint inhibitor[Title/Abstract])) OR (checkpoint blockade[Title/Abstract])) OR (immune checkpoint blocker[Title/Abstract])) OR (Programmed Cell Death 1[Title/Abstract])) OR (PD-1[Title/Abstract])) OR (PD 1[Title/Abstract])) OR (PD L1[Title/Abstract])) OR (PD-L1[Title/Abstract])) OR (CTLA-4[Title/Abstract])) OR (CTLA 4[Title/Abstract])) OR (immunotherapy[Title/Abstract])) OR (Nivolumab[Title/Abstract])) OR (Pembrolizumab[Title/Abstract])) OR (Atezolizumab[Title/Abstract])) OR (Avelumab[Title/Abstract])) OR (Durvalumab[Title/Abstract])) OR (Ipilimumab[Title/Abstract])) OR (Tremelimumab[Title/Abstract])) OR (Tecentriq[Title/Abstract])) OR (Sintilimab[Title/Abstract])) OR (Toripalimab[Title/Abstract])) OR (Camrelizumab[Title/Abstract])) OR (SHR-1210[Title/Abstract])) OR (Tislelizumab[Title/Abstract])) OR (Lambrolizumab[Title/Abstract])) OR (Keytruda[Title/Abstract])) OR (Cemiplimab[Title/Abstract])) OR (Sugemalimab[Title/Abstract])) OR (Serplulimab[Title/Abstract])) OR (adebrelimab[Title/Abstract])) OR (tiragolumab[Title/Abstract])

#8 #6 OR #7

#9 chemotherapy[Title/Abstract]

#10 ((((((((randomized controlled trial[Publication Type]) OR (controlled clinical trial[Publication Type])) OR (randomized[Title/Abstract])) OR (randomised[Title/Abstract])) OR (placebo[Title/Abstract])) OR (random*[Title/Abstract])) OR (randomly[Title/Abstract])) OR (trial[Title/Abstract])) OR (groups[Title/Abstract]) AND (humans[Filter])

#11 #5 AND #8 AND #9 AND #10

**Web of science**

#1 TS=("Esophageal Neoplasms" OR "esophageal neoplasm" OR "cancer of esophagus" OR "esophageal cancer" OR "esophageal cancers" OR "cancer of the esophagus" OR "esophagus cancer" OR "esophagus cancers" OR "ESCC" OR "esophageal squamous cell carcinoma")

#2 TS=(advanced OR metastatic OR unresectable OR inoperable)

#3 TS=("Immune Checkpoint Inhibitors" OR "immune checkpoint" OR "immune checkpoint inhibitor" OR "checkpoint blockade" OR "immune checkpoint blocker" OR

"Programmed Cell Death 1" OR "PD-1" OR "PD 1" OR "PD L1" OR "PD-L1" OR "CTLA-4" OR "CTLA 4" OR "immunotherapy" OR "Nivolumab" OR "Pembrolizumab" OR "Atezolizumab" OR "Avelumab" OR "Durvalumab" OR "Ipilimumab" OR "Tremelimumab" OR "Tecentriq" OR "Sintilimab" OR "Toripalimab" OR "Camrelizumab" OR "SHR-1210" OR "Tislelizumab" OR "Lambrolizumab" OR "Keytruda" OR "Cemiplimab" OR "Sugemalimab" OR "Serplulimab" OR "Adebrelimab" OR "Tiragolumab")

#4 TS="chemotherapy"

#5 TS=("randomized controlled trial" OR "controlled clinical trial" OR "clinical trial" OR "randomized" OR "randomised" OR "placebo" OR "random*" OR "randomly" OR "trial" OR "groups")

#6 #1 AND #2 AND #3 AND #4 AND #5

**EMBASE**

#1 "Esophageal Neoplasms" OR "esophageal neoplasm" OR "cancer of esophagus" OR "esophageal cancer" OR "esophageal cancers" OR "cancer of the esophagus" OR "esophagus cancer" OR "esophagus cancers" OR "ESCC" OR "esophageal squamous cell carcinoma"

#2 advanced OR metastatic OR unresectable OR inoperable

#3 "Immune Checkpoint Inhibitors" OR "immune checkpoint" OR "immune checkpoint inhibitor" OR "checkpoint blockade" OR "immune checkpoint blocker" OR "Programmed Cell Death 1" OR "PD-1" OR "PD 1" OR "PD L1" OR "PD-L1" OR "CTLA-4" OR "CTLA 4" OR "immunotherapy" OR "Nivolumab" OR "Pembrolizumab" OR "Atezolizumab" OR "Avelumab" OR "Durvalumab" OR "Ipilimumab" OR "Tremelimumab" OR "Tecentriq" OR "Sintilimab" OR "Toripalimab" OR "Camrelizumab" OR "SHR-1210" OR "Tislelizumab" OR "Lambrolizumab" OR "Keytruda" OR "Cemiplimab" OR "Sugemalimab" OR "Serplulimab" OR "Adebrelimab" OR "Tiragolumab"

#4 "chemotherapy"

#5 "randomized controlled trial" OR "controlled clinical trial" OR "clinical trial" OR "randomized" OR "randomised" OR "placebo" OR "random*" OR "randomly" OR "trial" OR "groups"

#6 #1 AND #2 AND #3 AND #4 AND #5 AND ([controlled clinical trial]/lim OR [randomized controlled trial]/lim)

**Cochrane Library**

#1 MeSH descriptor: [Esophageal Neoplasms] this term only

#2 (esophageal neoplasm* OR esophagus cancer* OR esophageal cancer* OR ESCC OR "esophageal squamous cell carcinoma"):ti,ab,kw

#3 #1 OR #2

#4 (advanced OR metastatic OR unresectable OR inoperable):ti,ab,kw

#5 #3 AND #4

#6 MeSH descriptor: [Immune Checkpoint Inhibitors] this term only

#7 (immune checkpoint* OR checkpoint blockade OR PD-1 OR PD-L1 OR CTLA-4 OR immunotherapy OR Nivolumab OR Pembrolizumab OR Atezolizumab OR Avelumab OR Durvalumab OR Ipilimumab OR Tremelimumab OR Tecentriq OR Sintilimab OR Toripalimab OR Camrelizumab OR SHR-1210 OR Tislelizumab OR Lambrolizumab OR Keytruda OR Cemiplimab OR Sugemalimab OR Serplulimab OR adebrelimab OR tiragolumab):ti,ab,kw

#8 #6 OR #7

#9 chemotherapy:ti,ab,kw

#10 #5 AND #8 AND #9

#11 #10 in Trials
